# Imaging characteristics of myocarditis after mRNA‐based COVID‐19 vaccination: a meta‐analysis

**DOI:** 10.1002/ehf2.14236

**Published:** 2022-11-20

**Authors:** Shingo Kato, Nobuyuki Horita, Daisuke Utsunomiya

**Affiliations:** ^1^ Department of Diagnostic Radiology, Graduate School of Medicine Yokohama City University Yokohama Japan; ^2^ Chemotherapy Center, Graduate School of Medicine Yokohama City University Yokohama Japan

Myocarditis following COVID‐19 mRNA vaccination is a rare adverse reaction with an incidence rate of approximately 0.0011%.^1^ Cardiac magnetic resonance imaging (CMR) allows for non‐invasive assessment of myocardial tissue characterization, including myocardial oedema and fibrosis, and has high diagnostic accuracy for diagnosing acute myocarditis.^2^ A recent study demonstrated the MRI characteristics of myocarditis after mRNA vaccination, slightly different from other‐cause of myocarditis; the most frequent location of late gadolinium enhancement (LGE) was the epicardial side of the basal inferolateral wall.^3^ Because the frequency of abnormalities on CMR is not well understood, we have performed a literature search and meta‐analysis to evaluate the imaging characteristics of myocarditis after mRNA vaccination on CMR.

On 1 February 2022, a literature search was performed using PubMed, Web of Science, the Cochrane library, and Embase by the search term ‘COVID‐19’, ‘SARS‐CoV‐2’, ‘vaccine’, ‘myocarditis’, and ‘MRI’. We identified 12 articles, including 274 patients with mRNA vaccine‐related myocarditis. We did not obtain IRB approval as this study is a meta‐analysis.

The majority of patients are young male recipients after the 2nd dose of the mRNA vaccine (median age: 17 years, male: 91.6%, after 2nd dose: 91.4%). The pooled prevalence according to the meta‐analysis using the inverse variance method and random‐model were as follows: the presence of LGE on the left ventricle (myocardial necrosis or fibrosis), 88% [95% CI (81%, 92%)], *I* ^2^ = 18%, (11 articles, 250 patients); the presence of LGE at the epicardial side, 76% [95% CI (61%, 91%)], *I* ^2^ = 68%, (8 articles, 82 patients); the presence of LGE at the lateral or inferolateral wall, 74% [95% CI (55%, 94%)], *I* ^2^ = 50%, (4 articles, 30 patients). While the rate of epicardial LGE on the lateral LV wall is high, the amount of the LGE is minimal (1–3.9% of total LV mass), and LV systolic function is almost normal (median LV ejection fraction: 58.3%, range: 51.6–60.6%). Other MRI findings are as follows: myocardial injury (T1 abnormality), 64% [95% CI (44%, 84%)], *I* ^2^ = 85%, (3 articles, 37 patients); myocardial oedema (T2 abnormality), 79% [95% CI (65%, 95%)], *I* ^2^ = 85%, (9 articles, 217 patients); pericardial enhancement, 71% [95% CI (15%, 100%)], *I* ^2^ = 91%, (2 articles, 26 patients); pericardial effusion 25% [95% CI (8%, 41%)], *I* ^2^ = 85%, (5 articles, 52 patients). Most of the patients meet the MRI‐based diagnostic criteria for acute myocarditis (Lake Louise criteria)^2^ {87% [95% CI (73%, 100%)], *I* ^2^ = 87%, (9 articles, 227 patients)} (*Figure* 1; *Table* 1).

**Figure 1:**
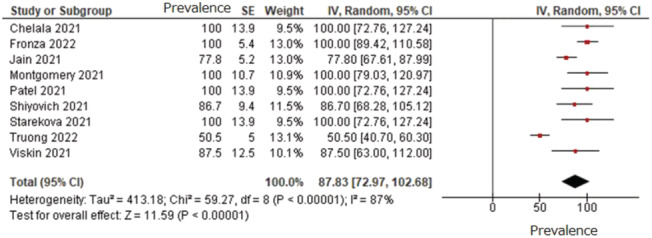
Prevalence of MRI‐based diagnostic criteria for acute myocarditis (Lake Louise criteria) in patients with mRNA vaccine‐related myocarditis. The prevalence of MRI‐based diagnostic criteria for acute myocarditis is 87% [95% CI (73%, 100%)], *I* ^2^=87%, (9 articles, 227 patients). MRI, magnetic resonance imaging

The above meta‐analyses clarified the imaging characteristics of the ‘unknown myocardial abnormalities’ regarding myocarditis after mRNA‐based COVID‐19 vaccination. Our analysis demonstrated that more than 80% of the patients had LGE on the LV myocardium, mainly located at the epicardial side of the lateral wall. In addition, abnormal T1 was found in 63%, and abnormal T2 was found in 79%; Lake Louise criteria were positive for 87% of the patients. Although the frequency of abnormalities on CMR is high, the severity of imaging findings is not severe, as reflected by low LGE volume (1–3.9%).^3^, ^4^ Furthermore, the short‐term outcome of vaccine‐related myocarditis is favourable. These facts will assure the doctors and vaccine recipients. However, long‐term consequences are still unknown as the presence of LGE is a strong predictor of adverse cardiac events in patients with other‐cause of myocarditis.^5^ Therefore, follow‐up might be necessary for patients with positive LGE. Further, a large‐scale prospective study is warranted to elucidate the long‐term outcome of LGE‐positive patients. We believe that our data will provide new insight into the diagnosis and clinical management of patients with vaccine‐related myocarditis.

## Sources of funding

The study received research grant from the Japan Society for the Promotion of Science: Grant‐in‐Aid for Early‐Career Scientists.

**Table 1:** Characteristics of included studies

Study	Number of patients	Age, (years)	Male, (%)	Symptomatic, (%)	LVEF, (%)	RVEF, (%)	LGE presence, (%)	LGE volume (%)	High native T1, (%)	High T2, (%)	Lake Louise criteria positive, (%)	Pericardial enhancement, (%)
Chelala 2021	5	17.1 ± 1.1	5/5 (100%)	N/A	56.2	54	100	NA	2/3 (67%)	67	5/5 (100%)	N/A
Das 2021	25	15.3 ± 1.5	22/25 (88%)	25/25 (100%)	N/A	N/A	94	N/A	N/A	37.5	NA	N/A
Dionne 2021	15	15 (12–18)	14/15 (93%)	15/15 (100%)	58.6	N/A	80	N/A	N/A	N/A	NA	N/A
Fronza 2022	21	31 ± 14	17/21 (81%)	21/21 (100%)	58	54	81	1 (0–2)	14/21 (67%)	79	21/21 (100%)	9/21 (45%)
Jain 2021	63	15.6 ± 1.8	58/63 (92%)	63/63 (100%)	58	N/A	88	N/A	N/A	89	49/63 (88%)	N/A
Marshall 2021	7	16.7 ± 1.6	7/7 (100%)	7/7 (100%)	N/A	N/A	100	N/A	N/A	100	NA	N/A
Montgomery 2021	8	25 (20–51)	23/23 (100%)	N/A	N/A	N/A	100	N/A	N/A	100	8/8 (100%)	N/A
Patel 2021	5	24.6 ± 7.3	5/5 (100%)	5/5 (100%)	60.6	N/A	80	N/A	N/A	100	5/5 (100%)	N/A
Shiyovich 2021	15	32 (22.5–40)	15/15 (100%)	13/15 (87%)	60.2	N/A	87	3.9 ± 4.5	6/13 (46%)	0	0/15 (0%)	N/A
Starekova 2021	5	25.2 ± 9.3	4/5 (80%)	5/5 (100%)	51.6	53.2	100	N/A	N/A	100	5/5 (100%)	5/5 (100%)
Truong 2022	97	15.8 (14.5–17.0)	126/139 (90.6)	138/139 (99.3%)	60	57.3	76	N/A	N/A	55.7	49/97 (50.5%)	N/A
Viskin 2021	8	20–34	N/A	N/A	N/A	N/A	N/A	N/A	N/A	N/A	7/8 (88%)	N/A

LGE, late gadolinium enhancement; LVEF, left ventricular ejection fraction; N/A, not applicable; RVEF, right ventricular ejection fraction.

Data are expressed as mean ± standard deviation, median (interquartile range), or number (%).
